# Resveratrol Improved Flow-Mediated Outward Arterial Remodeling in Ovariectomized Rats with Hypertrophic Effect at High Dose

**DOI:** 10.1371/journal.pone.0146148

**Published:** 2016-01-06

**Authors:** Marie Petit, Anne-Laure Guihot, Linda Grimaud, Emilie Vessieres, Bertrand Toutain, Marie-Claude Menet, Valérie Nivet-Antoine, Jean-François Arnal, Laurent Loufrani, Vincent Procaccio, Daniel Henrion

**Affiliations:** 1 University of Angers, Angers, France; 2 CNRS UMR-6214, Angers, France; 3 INSERM UMRS-1083, Angers, France; 4 CARFI (Cardiovascular Function In vitro) facility, Angers, France; 5 UMR-S1140, Faculty of Pharmacy, Paris Descartes University, Paris, France, and Assistance Publique Hôpitaux de Paris, Department of Biochemistry, Georges Pompidou European Hospital, Paris, France; 6 UMR-S1144, Faculty of Pharmacy, Paris Descartes University, Paris, France, and Assistance Publique Hôpitaux de Paris, Department of Biochemistry, Cochin Hospital, Paris, France; 7 University hospital (CHU) of Angers, Angers, France; 8 INSERM U1048, Toulouse III Paul Sabatier University, University hospital of Toulouse, Toulouse, France; The Chinese University of Hong Kong, HONG KONG

## Abstract

**Objectives:**

Chronic increases in blood flow in resistance arteries induce outward remodeling associated with increased wall thickness and endothelium-mediated dilatation. This remodeling is essential for collateral arteries growth following occlusion of a large artery. As estrogens have a major role in this remodeling, we hypothesized that resveratrol, described as possessing phytoestrogen properties, could improve remodeling in ovariectomized rats.

**Methods:**

Blood flow was increased *in vivo* in mesenteric arteries after ligation of adjacent arteries in 3-month old ovariectomized rats treated with resveratrol (5 or 37.5 mg/kg per day: RESV5 or RESV37.5) or vehicle. After 2 weeks arterial structure and function were measured *in vitro* in high flow (HF) and normal flow (NF) arteries isolated from each rat.

**Results:**

Arterial diameter was greater in HF than in NF arteries in ovariectomized rats treated with RESV5 or RESV37.5, not in vehicle-treated rats. In mice lacking estrogen receptor alpha diameter was equivalent in HF and NF arteries whereas in mice treated with RESV5 diameter was greater in HF than in NF vessels. A compensatory increase in wall thickness and a greater phenylephrine-mediated contraction were observed in HF arteries. This was more pronounced in HF arteries from RESV37.5-treated rats. ERK1/2 phosphorylation, involved in hypertrophy and contraction, were higher in RESV37.5-treated rats than in RESV5- and vehicle-treated rats. Endothelium-dependent relaxation was greater in HF than in NF arteries in RESV5-treated rats only. In HF arteries from RESV37.5-treated rats relaxation was increased by superoxide reduction and markers of oxidative stress (p67phox, GP91phox) were higher than in the 2 other groups.

**Conclusion:**

Resveratrol improved flow-mediated outward remodeling in ovariectomized rats thus providing a potential therapeutic tool in menopause-associated ischemic disorders. This effect seems independent of the estrogen receptor alpha. Nevertheless, caution should be taken with high doses inducing excessive contractility and hypertrophy in association with oxidative stress in HF arteries.

## Introduction

The arterial tree has an important plasticity, which allows adapting to continuous changing conditions. Structural remodeling involves the rearrangement of the components of the vascular wall [1] whereas functional remodeling is characterized by changes in the relative importance of constrictor and dilator pathways [2]. Resistance arteries play a major role in the control of local blood flow to organs and their dysfunction is associated to the major vascular diseases [3]. They are sensitive to chronic changes in the hemodynamic environment and undergo rapid structural and functional remodeling [4–6]. Chronic increases in blood flow (shear stress) induce outward remodeling in resistance arteries associated with a functional remodeling mainly characterized by improvement of endothelium (NO)-dependent dilation [7–12]. Chronic increases in blood flow occur in physiological situations such as growth, pregnancy or physical exercise [6, 13]. In pathological conditions, a chronic increase in blood flow is expected in resistance arteries feeding ischemic tissues [3, 5, 6]. Indeed, high-flow-mediated outward remodeling allows collateral arteries growth and thus it is essential in post-ischemic revascularization besides angiogenesis [14, 15].

The ability of resistance arteries to enlarge their diameter in response to a chronic increase in blood flow in vivo is strongly reduced in rat models of aging [16–18], hypertension [19, 20] and diabetes [11, 21, 22] although maintained in obesity [23]. Flow-mediated outward remodeling does not occur in male rats aged 10 months or more [16, 17, 24, 25] whereas it is maintained in female rats aged 12 to 18 months [26]. Moreover, we have shown that flow-mediated outward remodeling does not take place in mice lacking the estrogen receptor alpha [27].

Epidemiological studies have demonstrated that women, before menopause, are better protected than men against many cardiovascular diseases [28]. The decline in ovarian function is associated with decreased NO production [29] and stimulation of the NO-pathway explains, at least in part, the protective effect of estrogens on the vascular wall [30, 31]. Nevertheless, following the WHI (Women Health Initiative) study estrogen therapy for menopaused women failed to demonstrate beneficial effect [32]. Consequently, phytoestrogen therapy is now widely used although its efficiency still remains a matter of debate [33]. Resveratrol has been shown to induce NO production by activating the ERalpha-Src-caveolin-1 pathway in HUVECs [34]. Nevertheless, resveratrol activates other molecular targets, especially in the vascular endothelium [35]; many of them being also involved in flow-mediated outward hypertrophic remodeling [6]. Thus we aimed at testing the hypothesis that resveratrol could activate flow-mediated remodeling. In order to test this hypothesis, we used ovariectomized female rats submitted to a local and chronic increase in blood flow in mesenteric arteries in vivo [9, 27]. Since the bioavailability and metabolism of resveratrol [36] has been a matter of debate, we used subcutaneous osmotic minipumps to deliver continuously trans-resveratrol.

## Material and Methods

### Animal Protocol

Three-month old female Wistar rats (Charles River France) were ovariectomized (OVX) as previously described [37] under isoflurane anesthesia (2.5%). After 1 week, rats were anesthetized (isoflurane, 2.5%) and submitted to surgery in order to increase blood flow in one mesenteric artery as previously described [38]. Briefly, 3 consecutive first-order arteries were used. Ligatures were applied to second-order branches downstream the first and third first-order arteries as shown on S1 Fig.

In an independent series of experiments, mice lacking the gene encoding for the estrogen receptor alpha and their littermate wild-type controls were submitted to a similar protocol as previously described [27].

Animals were treated with buprenorphine (Temgesic^®^; 0.1 mg/kg, s.c.) before and after surgery. They were housed in a thermoregulated pre-warmed, humidified incubator allowing animal surveillance. The artery located between these two ligated arteries was designed as high flow (HF) artery. Arteries located at distance of the ligated arteries were used as normal flow (NF), i.e. control vessels [38].

Ovariectomized rats were randomly treated, using Alzet® osmotic minipumps, with trans-resveratrol (3,5,4′-trihydroxystilbene, Sigma, 5 or 37.5mg/kg per day, 2 weeks, diluted in dimethylsulfoxide, DMSO, 7% or 50% respectively, n = 12 rats per groups) or with the vehicle alone (DMSO, 50% n = 12 rats). Minipumps were implanted subcutaneously under isoflurane anesthesia (2.5%). Rats were treated with buprenorphine (Temgesic^®^; 0.1 mg/kg, s.c.) before and after any surgery.

After 14 days, rats were anaesthetized (isoflurane 2.5%) and arterial blood pressure measured in the carotid artery [39]. They were then sacrificed in a CO_2_ chamber. The mesentery was quickly removed and placed in an ice-cold physiological salt solution (PSS) of the following composition (mM): 130, NaCl; 15, NaHCO_3_; 3.7, KCl; 1.2 KH_2_PO_4_; 1.2, MgSO_4_; 11, glucose; 1.6, CaCl_2_; and 5, HEPES, pH 7.4, PO_2_ 160 mmHg, PCO_2_ 37 mmHg. Mesenteric arteries (HF and NF) were gently dissected and divided into two segments, proximal for the functional study and distal for histological and biochemical studies. A segment of the liver was also collected and quickly frozen before measurement of its resveratrol content.

The investigation was performed in agreement with the guidelines from Directive 2010/63/EU of the European Parliament on the protection of animals used for scientific purposes (authorization of the laboratory # 00577). The protocol was approved by the Institutional Animal Care and Use Committee (IACUC) of the Pays de La Loire (“*Comité d’éthique en experimentation animale des Pays de la Loire*”: http://www.ceea-paysdelaloire.com/), under the protocol number: CEEA PdL # 2008.10.

### Diameter and structure of mesenteric arteries *in vitro*

Segments of HF and NF arteries were cannulated at both ends, mounted in a pressure arteriograph (LSI, Burlington, VT) [40] and bathed in a Ca^2+^-free PSS containing EGTA (2 mmol/L) and sodium nitroprusside (SNP, 10 μmol/L). Arterial diameter was measured in response to stepwise increase in intraluminal pressure (10 to 125 mmHg) and data recorded using Acqknoledge® (Biopac) [41]. Pressure was then set at 75 mmHg and arteries were fixed in formaldehyde in order to measure media cross-section area and wall thickness as previously described [42].

### Pharmacological profile of isolated NF and HF arteries

Other arterial segments (2-mm long each) were dissected and mounted in a wire myography (DMT) [43]. Cumulative concentration-response curves to phenylephrine (0.001 to 10μmol/L) and acetylcholine (0.01 to 10 μmol/L) and SNP (0.01 to 10 μmol/L) were performed. Cumulative concentration-response curves to acetylcholine were obtained before and after incubation (20 minutes) with the NO-synthase inhibitor L-NAME (10μmol/L) or with superoxide dismutase mimetic 1-Oxyl-2,2,6,6-tetramethyl-4-hydroxypiperidine (tempol, 10 μmol/L, 20 min) plus catalase (80 U/mL, 20 min). Acetylcholine- and SNP- dependent relaxation was performed after precontraction of the arteries with phenylephrine to 70% of their maximal contractile response [44].

### Western blot analysis

Segments of HF and NF mesenteric arteries were collected, quickly frozen, and then pulverised in liquid nitrogen. The sample powders obtained were resuspended in lysis buffer. Vessel extracts were incubated on ice for 30 minutes and then centrifuged (14,000 rpm, 20 minutes at 4°C).

Proteins (30 μg total protein from each sample) were separated by 10% SDS-PAGE and transferred to nitrocellulose. Membranes were incubated with the primary antibody (Biosciences, eNOS, 1:1000; p67phox, 1/500; gp91phox, 1/500; MnSOD, 1/1000; Cu/ZnSOD, 1/1000; COX IV (Cytochrome C Oxidase IV), 1/1000; sirtuin-1, 1/500; cytochrome C, 1/1000; pGc1alpha (Peroxisome proliferator-activated receptor gamma coactivator 1-alpha), 1/1000; ERK1 and ERK2, 1/1000; phospho ERK1 and phosphoERK2, 1/1000; COX1, 1/1000; COX2, 1/5000; beta-actin, 1/2000), and incubated with horseradish peroxidase-conjugated secondary antibody (Amersham) at room temperature. Proteins were visualised using the ECL-Plus Chemiluminescence kit (Amersham) [10].

### Measurement of liver resveratrol concentration

Liver samples were prepared as previously described [45]. Briefly, an aliquot (50 mg) of liver was crushed by Precelly^R^24 (Ozyme) with 1 mL of MeOH/H_2_O/acetic acid (80/20/0.5; V/V/V), let stand for 20 min and centrifuged at 14000 g for 5 min. Supernatants were separated and evaporated to dryness by using a Speed Vac Concentrator (Savant, SPD131DDA, Thermo Fischer Scientific, Les Ulis, France). The dried extracts were reconstituted in 100 μL ACN/water/Formic Acid (20/80/0.1; V/V/V). Resveratrol concentration in the extracts was determined as previously described [46] using liquid chromatography followed by mass spectrometry. Samples were injected into an Acquity UPLC system (Waters, Manchester, U.K.), equipped with an Acquity BEH C18 pre-column (particle size 1.7 μm) and an Acquity UHPLC BEH C18 column (50 mm × 2.1 mm inner diameter, particle size 1.7 μm) and which were purchased from Waters (Guyancourt, France). The auto-sampler of the Acquity UPLC was kept at+15°C. The mobile phase was a mixture of water (solvent A) and ACN (solvent B) in gradient mode. Both solvents were degassed by the integrated Acquity UPLC degasser. The entire UHPLC effluent entered the electrospray ionization chamber. Mass spectra were recorded with a Waters Synapt G2 HDMS mass spectrometer (Waters, Manchester, U.K.). Measurements were performed using negative electrospray ionization (ESI) in full scan data acquisition mode. Data processing was performed using the MassLynx software (V 4.1, SCN 779, Waters, Manchester, UK).

### Statistical Analysis

Results were expressed as means ± SEM. Significance of the differences between groups was determined using ANOVA for consecutive measurements for pressure-diameter curves and for the concentration-response curves to phenylephrine, acetylcholine and SNP followed by a Bonferroni’s test. For other data and Table 1 a one-way ANOVA test was performed followed by a Bonferroni’s test. Statistical analysis was performed using GraphPad®. Values less than 0.05 were considered significant.

**Table 1:** Characteristics of experimental animals.

	Vehicle	resveratrol5.0 mg/kg	resveratrol37.5 mg/kg
Body weight (g)	276 ± 4	268 ± 10	279 ± 11
MAP (mmHg)	85.7 ± 2	87.5 ± 3	90.7 ± 2
Uterus weight (mg)	0.21±0.05	0.23±0.05	0.27±0.06
Liver resveratrol (nmol/g)	< 0.2	2.86±0.64*	11.20±1.56*#
n	10	9	9

Body weight, mean arterial blood pressure (MAP), uterus weight and liver resveratrol content measured in ovariectomized rats treated with resveratrol 5.0 or 37.5mg/kg or with the solvent. Mean ± sem is presented.

*P<0.05, versus vehicle.

^#^P<0.05, resveratrol 37.5 mg/kg versus resveratrol 5.0 mg/kg.

## Results

### Characteristics of experimental animals

Body weight, mean arterial blood pressure and uterus weight were not significantly affected by the treatment with resveratrol or by the vehicle (Table 1).

Resveratrol liver concentration was higher in rats treated with RESV37.5 than in rats treated with RESV5. Both were significantly higher than in vehicle-treated rats (Table 1).

### Passive arterial diameter

**Fig 1:**
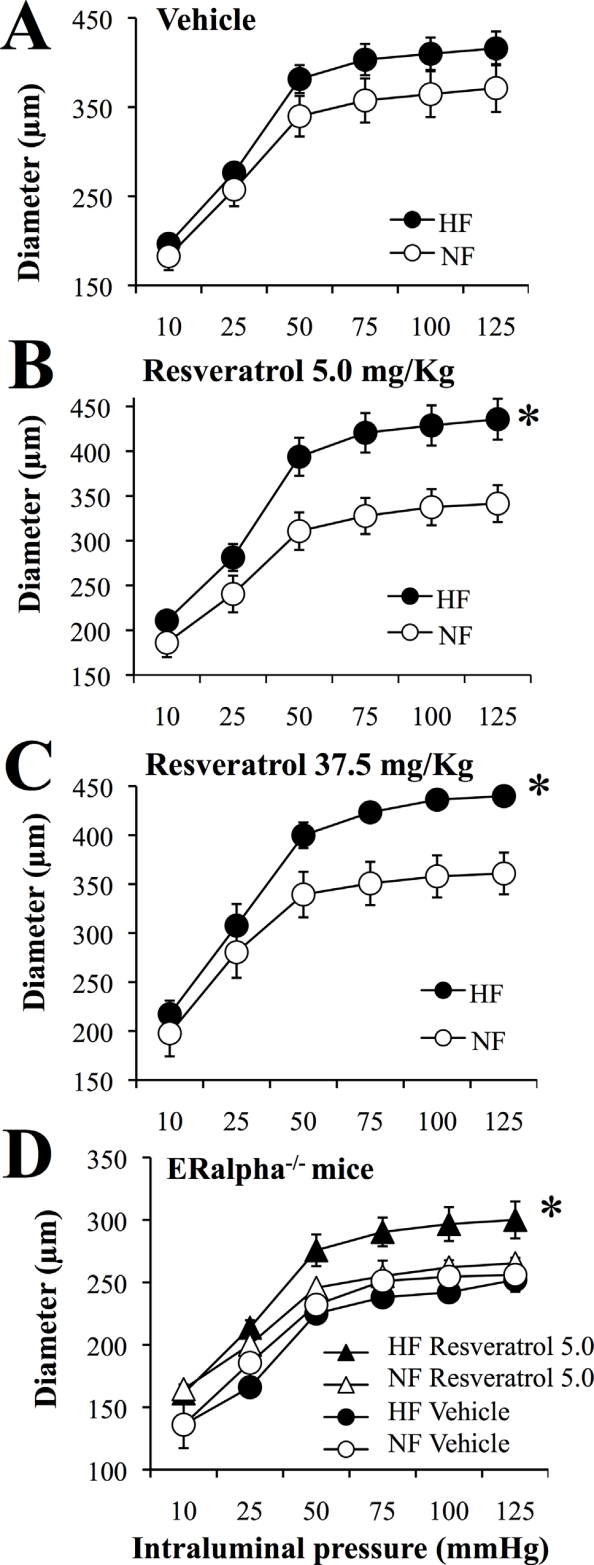
Arterial diameter in mesenteric arteries submitted to high blood flow. Luminal diameter was measured in mesenteric arteries submitted to a chronic increase in blood flow (high flow: HF) and in control arteries submitted to normal flow (NF). Arteries were isolated from ovariectomized rats treated with resveratrol 5.0 (B, n = 9 rats) or 37.5mg/kg (C, n = 9 rats) or with the vehicle (A, n = 10 rats). In a separate series of experiments, ERalpha-/- mice were ovariectomized and treated with the solvent or with resveratrol 5mg/kg (D, n = 4 mice per group). Mean ± sem is represented. *P<0.05, HF versus NF arteries.

Two weeks after arterial ligation, arterial diameter was determined in vitro in response to intraluminal pressure ranging from 10 to 125 mmHg (Fig 1A–1D). Passive arterial diameter was significantly higher in HF than in NF arteries in resveratrol 5.0 mg/kg (RESV5) (Fig 1B) and resveratrol 37.5 mg/kg (Fig 1C) treated rats. In vehicle-treated rats, the diameter of the HF arteries was not significantly different from that of NF arteries (Fig 1A).

In mice lacking the gene encoding for ERalpha and treated with RESV5, the diameter of the HF arteries was significantly greater than that of NF vessels. By contrast, HF and NF arteries diameter was equivalent in solvent-treated mice in accordance with our previous experiments [27] in ERalpha-/- mice (Fig 1D).

### Arterial structure and contractility

**Fig 2:**
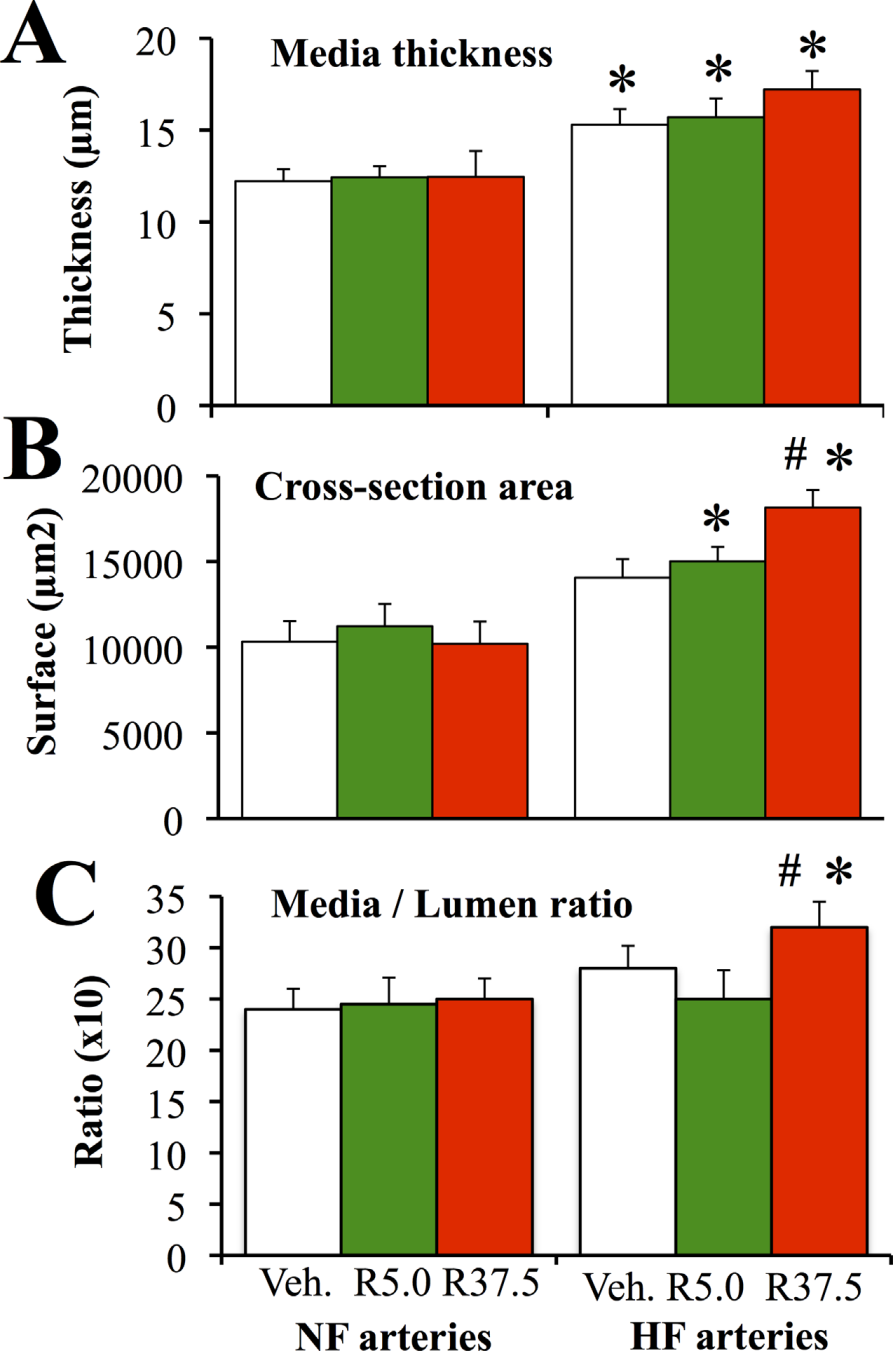
Arterial structure in mesenteric arteries submitted to high blood flow. Arterial media thickness (A), media cross-section area (B) and media to lumen ratio (C) were measured in mesenteric arteries submitted to a chronic increase in blood flow (high flow: HF) and in control arteries submitted to normal flow (NF). Arteries were isolated from ovariectomized rats treated with resveratrol 5.0 (R5.0 or Resv. 5.0, n = 9 rats) or 37.5mg/kg (R37.5 or Resv. 37.5, n = 9 rats) or with the vehicle (Veh., n = 10 rats). Mean ± sem is represented. *P<0.05, HF versus NF arteries. ^#^P<0.05, R37.5 versus vehicle.

Arterial media thickness and cross-section area were greater in HF than in NF arteries in RESV5-, RESV37.5- and vehicle-treated rats (Fig 2A and 2B). Cross-section area in HF arteries was higher in RESV37.5-treated rats than in RESV5- and vehicle-treated rats (Fig 2B). Consequently, wall to lumen ratio was also higher in RESV37.5-treated rats than in RESV5- and vehicle-treated rats (Fig 2C).

**Fig 3:**
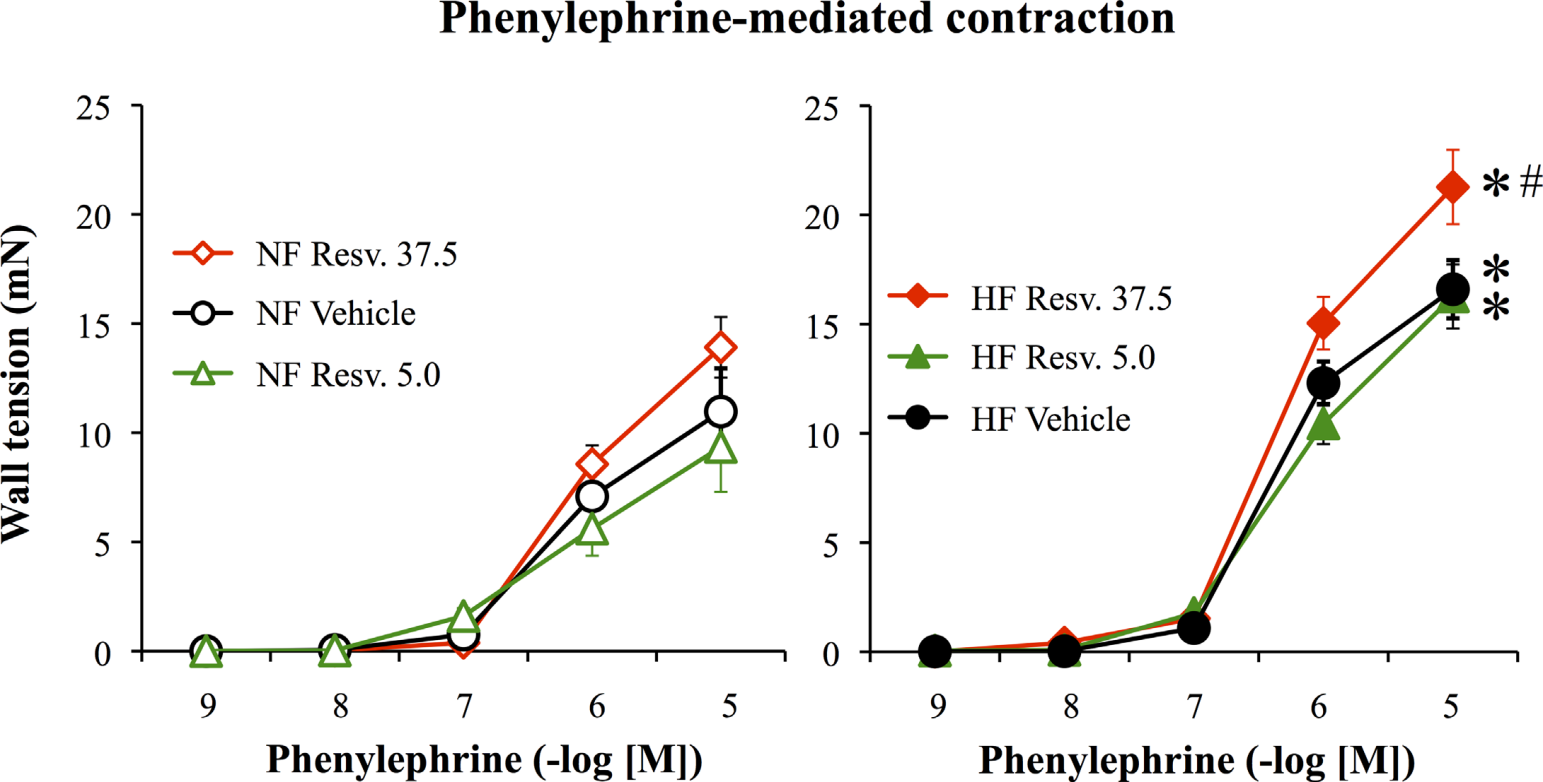
Phenylephrine-mediated contraction in mesenteric arteries submitted to high blood flow. Phenylephrine-mediated contraction was measured in mesenteric arteries submitted to a chronic increase in blood flow (high flow: HF, right panel) and in control arteries submitted to normal flow (NF, left panel). Arteries were isolated from ovariectomized rats treated with resveratrol 5.0 (R5.0 or Resv. 5.0, n = 9 rats) or 37.5mg/kg (R37.5 or Resv. 37.5, n = 9 rats) or with the vehicle (n = 10 rats). Mean ± sem is represented. *P<0.05, HF versus NF arteries. ^#^P<0.05, R37.5 versus vehicle.

Phenylephrine induced a concentration-dependent contraction in rat mesenteric arteries. This contraction was greater in HF than in NF arteries in the 3 study groups. Nevertheless, in HF arteries from RESV37.5-treated rats phenylephrine-mediated contraction was higher than in HF arteries from RESV5- and vehicle-treated rats (Fig 3). This was also observed with KCl (80 mmol/L)-mediated contraction which was greater in HF arteries from RESV37.5-treated rats (14.7±1.4 mN, n = 9, P<0.05) compared to HF arteries from RESV5-(10.6±0.9 mN, n = 9) and vehicle-treated rats (10.0±1.2 mN, n = 10).

### Endothelium-dependent and independent relaxation

**Fig 4:**
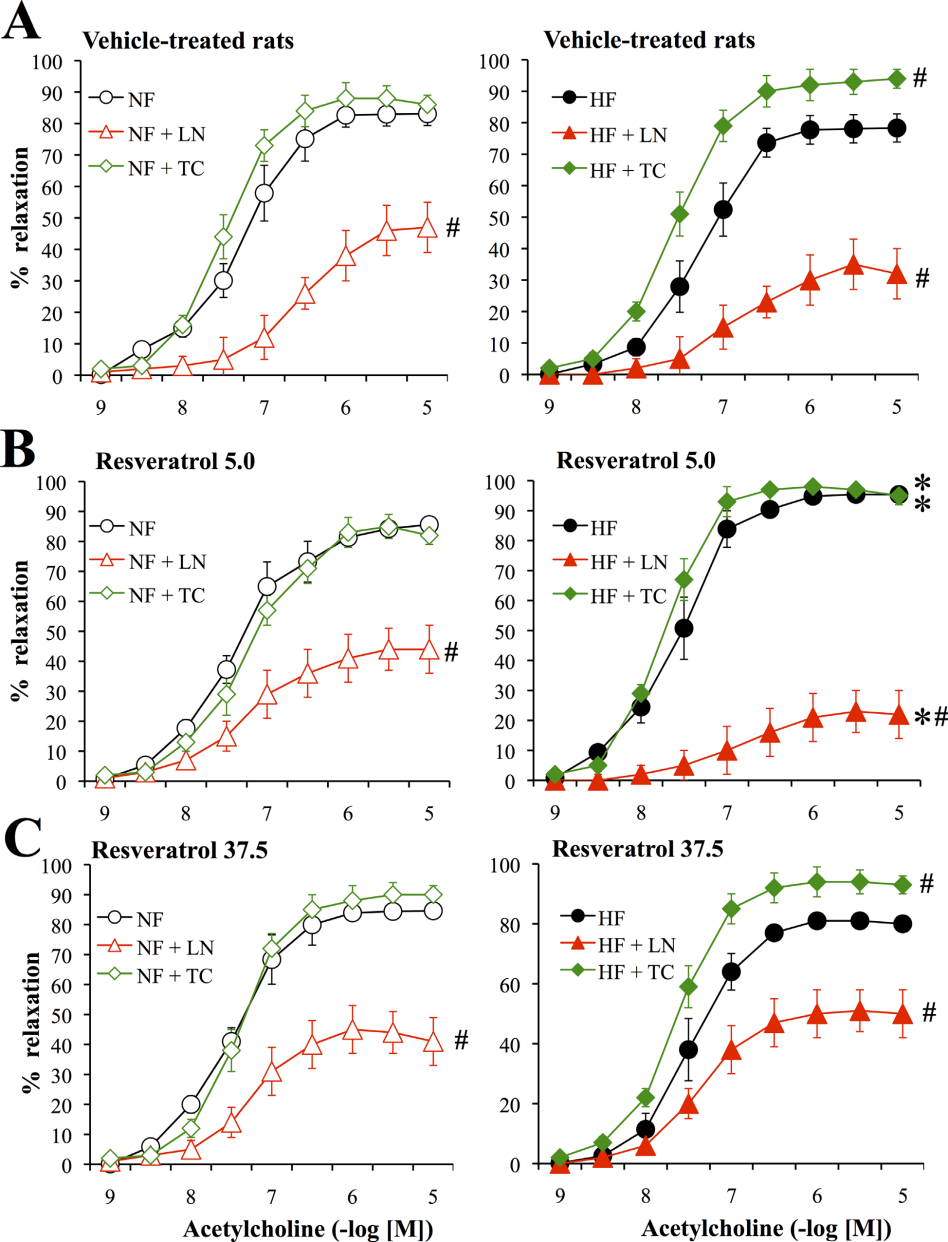
Acetylcholine-mediated relaxation in mesenteric arteries submitted to high blood flow. Concentration response curve to acetylcholine obtained in mesenteric arteries submitted to a chronic increase in blood flow (high flow: HF, right panel) and in control arteries submitted to normal flow (NF, left panel). Concentration response curve to acetylcholine were obtained before and after treatment of arteries with L-NAME (LN) or the combination of tempol plus catalase (TC). Arteries were isolated from ovariectomized rats treated with the vehicle (A, n = 10 rats), resveratrol 5.0mg/kg (B, n = 9 rats) or resveratrol 37.5mg/kg (C, n = 9 rats). Mean ± sem is represented. *P<0.05, HF versus NF arteries. ^#^P<0.05, effect of TC or LN (versus control without treatment).

Acetylcholine induced concentration-dependent relaxation in rat mesenteric arteries (Fig 4A–4C). Relaxation to acetylcholine was significantly greater in HF than in NF arteries in rats treated with RESV5.0 (Fig 4B). On the other hand, acetylcholine-mediated relaxation was not different in HF and NF vessels in vehicle- (Fig 4A) and RESV37.5-treated rats (Fig 4C).

L-NAME reduced acetylcholine-dependent relaxation in all group. The inhibitory effect of L-NAME was significantly more pronounced in HF arteries from RESV5.0-treated rats compared to NF vessels. This was not the case in the 2 other groups.

Tempol plus catalase did not significantly affect acetylcholine mediated-relaxation in NF arteries in the 3 groups. In HF arteries Tempol plus catalase increased acetylcholine-dependent relaxation in vehicle and RESV37.5-treated rats (Fig 4A and 4C). This was not observed in RESV5.0-treated rats (Fig 4B).

### Protein expression analysis

**Fig 5:**
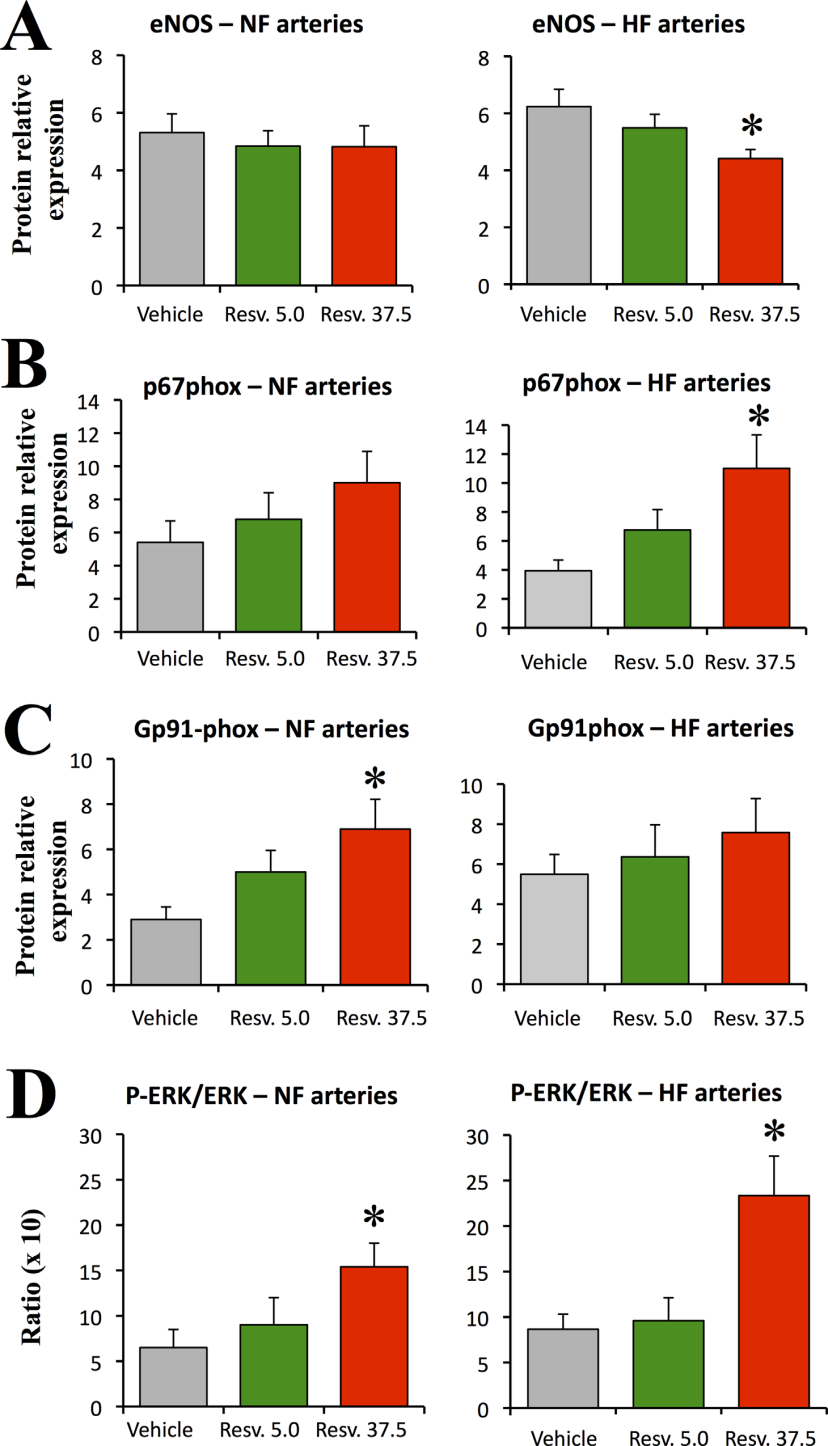
Expression level of eNOS (A), p67 phox (B), Gp91 phox (C) and ERK1/2 activation (D, ratio of phospho-ERK1/2 to ERK1/2) expression level. The ratio of protein expression in HF to the expression level in NF arteries is shown in the right panel. Arteries were isolated from ovariectomized rats treated with resveratrol 5.0 (Resv. 5.0, n = 9 rats) or 37.5mg/kg (Resv. 37.5, n = 9 rats) or with the vehicle (n = 10 rats). Mean ± sem is represented. Blots are shown in S3A–S3D Fig. *P<0.05, effect of the treatment: Resv. 5.0 or Resv. 37.5 versus vehicle.

The protein expression level of eNOS was not affected by the treatments in NF mesenteric arteries (Fig 5A, left panel). In HF arteries eNOS expression level was significantly reduced in RESV37.5-treated compared to vehicle-treated rats (Fig 5A, right panel).

The expression level of p67phox tended to be higher in the NF mesenteric artery in RESV37.5-treated rats than in vehicle-treated rats but the values did not reach significance. Nevertheless, in HF arteries p67phox expression level was significantly greater in RESV37.5-treated rats than in vehicle-treated rats (Fig 5B). The expression level of gp91phox was higher in NF mesenteric arteries in RESV37.5-treated rats than in vehicle-treated rats but not in HF arteries (Fig 5C). The expression level of MnSOD and Cu/Zn SOD and well as the expression level of COX1 and COX2 were not affected by the treatments in both NF and HF vessels (S2 Fig).

The ratio of phospho-ERK1/2 to ERK1/2 was significantly higher in RESV37.5-treated rats than in vehicle-treated rats in both NF and HF mesenteric arteries (Fig 5D).

**Fig 6:**
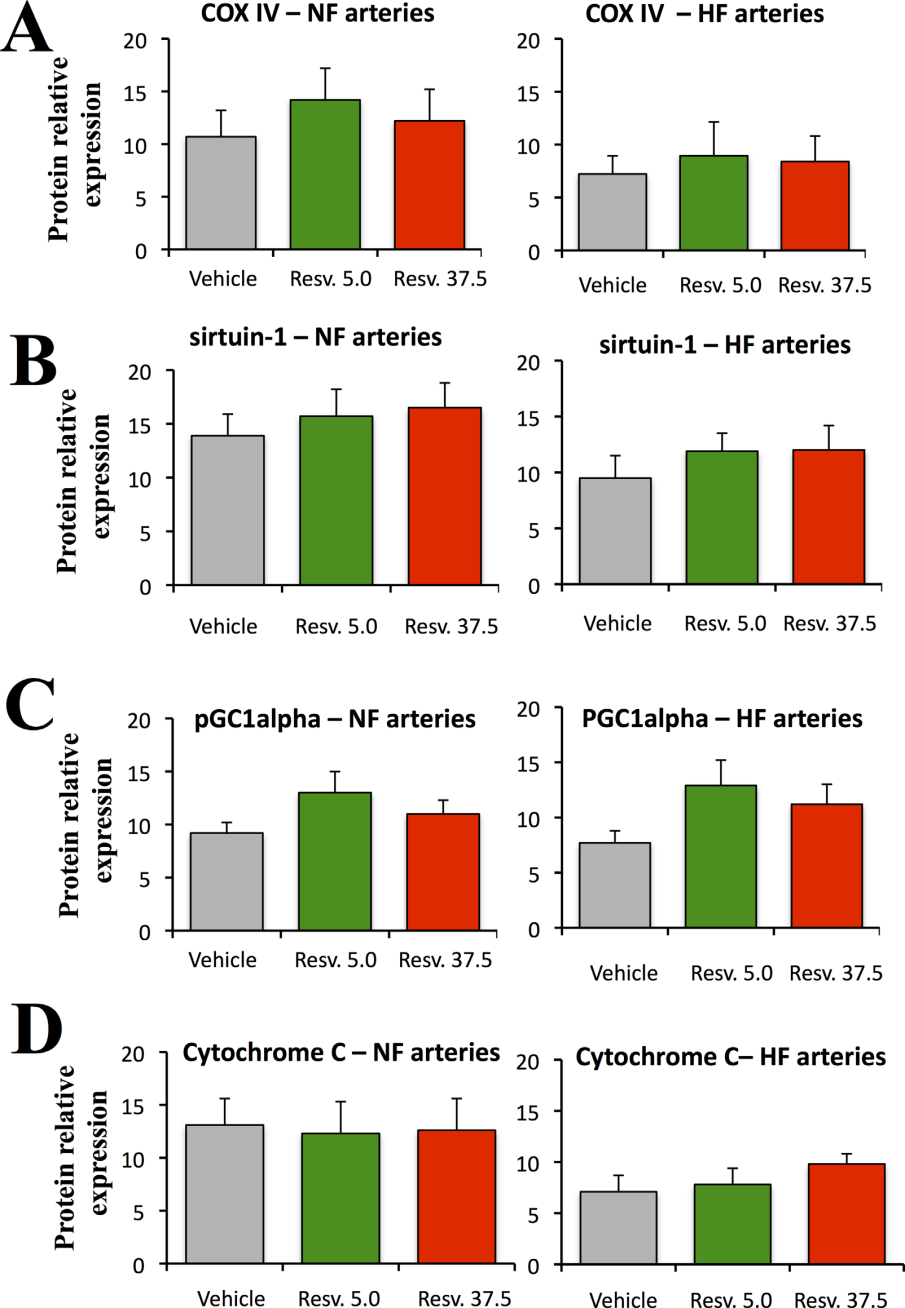
Mitochondrial proteins expression level. The expression level of of Cytochrome-C Oxidase IV (COX IV, A), sirtuin-1 (B), pGC1alpha (C), and Cytochrome C (D) was determined using Western-blot analysis in mesenteric arteries (left panel). The ratio of the expression level in HF to the expression level in NF arteries is shown in the right panel. Arteries were isolated from ovariectomized rats treated with resveratrol 5.0 (Resv. 5.0, n = 9 rats) or 37.5mg/kg (Resv. 37.5, n = 9 rats) or with the vehicle (n = 10 rats). Mean ± sem is represented (n = 12 rats per group). Blots are shown in S3A–S3D Fig. *P<0.05, effect of the treatment: Resv. 5.0 or Resv. 37.5 versus vehicle.

Cytochrome-C Oxidase IV, sirtuin-1, PGC1alpha and Cytochrome c expression level was not affected by the treatment with resveratrol (low and high dose) in both NF and HF mesenteric arteries (Fig 6).

Blots of the proteins analyzed above are shown in S3A–S3D Fig.

## Discussion

The main finding of the present study is that both a low dose and a high dose of resveratrol improved flow-mediated outward remodeling in ovariectomized rats. Nevertheless, the high dose of resveratrol induced oxidative stress, hypertrophy, and excessive contractility in the remodeled artery.

A chronic increase in blood flow induces a change in structure and function of resistance arteries. Besides the structural remodeling characterized by a diameter increase, a functional remodeling takes place with an increased responsiveness of the endothelium to vasodilator stimuli [8, 10, 47]. Flow-mediated structural and functional remodeling has a major role in ischemic diseases as it allows collateral arteries growth after occlusion of a large artery [14, 15]. In addition, this remodeling is associated with improved endothelium-mediated dilation [9, 11], which also contributes to increase tissue perfusion [14].

Estrogens exert beneficial effects on the vasculature [25] and flow-mediated remodeling of mesenteric resistance arteries dependent on the activation of the estrogen receptor alpha by estrogens [18, 26, 27]. Although women are better protected than men against cardiovascular diseases before menopause [28], the WHI study led to the interruption of estrogen substitution treatments in post-menopausal women [28]. Consequently, alternative therapies are now used without enough proofs of efficacy. Unfortunately, phytoestrogens such as soy derivatives or red clover extracts fail improving most of menopausal symptoms [48, 49]. Resveratrol, on the other hand, has been proposed as a potentially protective compound with vasodilator and antioxidant properties [50]. Nevertheless, the effect of resveratrol on flow-mediated remodeling did not involve the activation of ERalpha as it was preserved in ERalpha-/- mice. This finding provides a potential mechanism, at least in part, for some beneficial effects of this compound independent of ERalpha and thus potentially efficient in menopaused women without the estrogenic side effects highlighted by the WHI study.

The ovariectomy suppressed flow-mediated outward remodeling and reduced endothelium-mediated relaxation in the HF artery due to oxidative stress, as previously shown [18, 26, 27]. Resveratrol reversed this effect of ovariectomy although the effect of the 2 doses used was different as regard to endothelium-mediated relaxation and wall thickening in the HF artery. Indeed, with the high dose, which increased oxidative stress and ERK1/2 activation, the positive effect of resveratrol on endothelium-mediated relaxation was not observed and wall hypertrophy was found.

The low dose of resveratrol used in the present study restored diameter expansion in mesenteric arteries in ovariectomized rats and increased L-NAME-sensitive endothelium-dependent relaxation. This is in agreement with previous studies using similar animal models [9, 10]. This dose of resveratrol (5mg/kg per day) did not significantly affect markers of oxidative stress and the main effectors of mitochondrial bioenergetics. Thus, the main effect of the low dose of resveratrol was its capacity to promote flow-mediated outward remodeling, which is comparable to the effect of estrogens [18, 26, 27] although the effect was independent of the ERalpha. The mechanism involved remains to be elucidated.

Although the high dose of resveratrol (37.5mg/kg per day) also restored diameter expansion in HF arteries, it also induced hypertrophy and an excessive contractility of the HF vessel. Furthermore, the high dose of resveratrol reduced endothelium-dependent relaxation probably through superoxide anion production as suggested by the effect of tempol and catalase, which increased the relaxation in the HF artery. In addition, overexpression of gp91phox was observed in rats treated with the high dose of resveratrol. These observations suggest that increased oxidative stress occurred with the high dose of resveratrol. This could contribute to the reduction of NO-mediated relaxation in HF arteries as previously shown [10]. Such an oxidative stress has already been observed in the aorta in a previous study using this high dose of resveratrol [51].

The double effect of resveratrol with a protective effect at a low dose and a deleterious effect at a high dose is in agreement with our previous work showing a dual effect of red wine polyphenols on post-ischemic revascularization following femoral artery ligation in the rat, the low dose improving angiogenesis and the high dose reducing it [52].

The high dose of resveratrol induced an excessive activation of ERK1/2 in HF arteries. This might explain the hypertrophy as evidenced by the increased wall to lumen ratio in the HF vessels. Indeed, ERK1/2 activation is essential for the compensatory increase in wall mass accompanying flow-mediated outward remodeling [38]. This wall thickening allows normalizing tensile stress due to the diameter expansion [6]. Nevertheless, an increased wall to lumen ratio is a strong marker of cardiovascular risk [53] suggesting that the high dose of resveratrol might have deleterious consequences in the long term despite a positive effect on flow-mediated diameter expansion. The excessive oxidative stress might also take part to the hypertrophy as shown in various cardiovascular disorders [54]. The increased ERK1/2 activation and oxidative stress may also explain the increased contractility to phenylephrine observed in HF arteries from rats treated with the high dose of resveratrol.

Resveratrol has been shown to activate mitochondrial bioenergetics through the activation of sirtuins, which are a family of NAD-dependent deacetylases. Among those, sirtuin-1 is considered as an important regulator of metabolism, able to activate both mitochondrial biogenesis by transcriptional activation of the PGC1-related signaling pathway [55]. Although this effect is clear in various cell types [56], less evidence is available in blood vessels. Resveratrol has been shown to reduce reactive oxygen species production in the rat aorta and in cultured bovine aortic endothelial cells through sirtuin-1 and NADPH-oxidase pathway, without changes in expression level [57]. Nevertheless, we did not evidence any effect of resveratrol on the expression level of sirtuin-1 in mesenteric arteries but we cannot exclude a post-translational activation by resveratrol as described previously [58].

The high dose of resveratrol used in the present study is equivalent to doses used in clinical studies [59–61], although lower doses are also used in humans [51]. Whereas, the high dose restored flow-mediated outward remodeling it also induced arterial hypertrophy, which is potentially deleterious in the long-term. This observation is in agreement with previous studies showing that resveratrol in the long-term and in pathophysiological conditions may exert deleterious effects [51, 62]. We have previously shown that resveratrol has either beneficial or deleterious effects on insulin sensitivity and arterial physiology, depending on the age and diet of the animals. Indeed, in old mice fed with a high-protein diet, resveratrol increased systemic inflammation and aorta superoxide production in association with reduced aortic distensibility [51]. On the other hand, the low dose of resveratrol has been shown to activate the respiratory chain through functional increase in energetic metabolism without AMPK involvement [56] whereas high doses of resveratrol are associated with reduced mitochondrial oxidative metabolism [56, 63] leading to oxidative stress.

## Conclusion

The present work showed that resveratrol was able to restore flow-mediated outward remodeling in ovariectomized rat. Thus resveratrol might be a potential therapeutic tool in ischemic disorders requiring collateral artery growth. Nevertheless, caution should be taken as high doses of resveratrol induced excessive contractility and hypertrophy. High doses can be obtained with commercially available resveratrol or even sold over the counter as a nutritional supplement. Nevertheless, the bioavailability of resveratrol remains debated [36]. Thus, despite the effect of resveratrol on flow-mediated remodeling, it should certainly not be used primarily and not alone.

## Supporting Information

S1 Fig (PDF)

S2 Fig (PDF)

S3 Fig (PDF)
