# Supplementary material for: Resveratrol Improved Flow-Mediated Outward Arterial Remodeling in Ovariectomized Rats with Hypertrophic Effect at High Dose
Source: PLoS One. 2016 Jan 6;11(1):e0146148. doi: 10.1371/journal.pone.0146148 (PMC4703409; doi:10.1371/journal.pone.0146148)
Supplement: S1 Fig — (PDF) [file pone.0146148.s001.pdf]

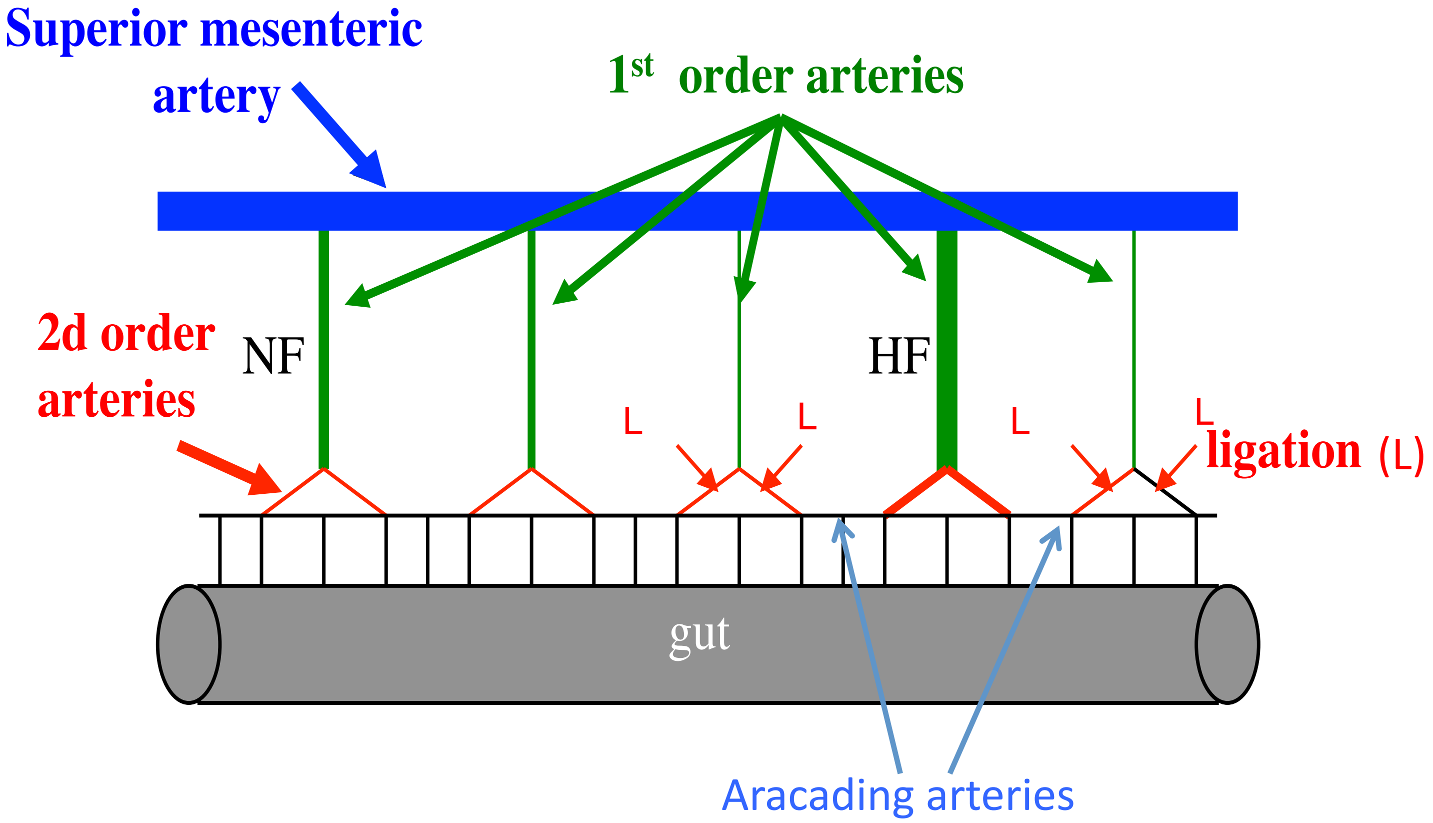

S1 Fig. Chronic increase in blood flow in vivo: scheme depicting the surgical procedure applied to the mesenteric arteries in order to increase locally blood flow in one artery (high flow: HF) after ligature of second order mesenteric arteries as shown by the red arrows (L). Arteries located at distance were used as control arteries (normal flow: NF).
