# Supplementary material for: Resveratrol Improved Flow-Mediated Outward Arterial Remodeling in Ovariectomized Rats with Hypertrophic Effect at High Dose
Source: PLoS One. 2016 Jan 6;11(1):e0146148. doi: 10.1371/journal.pone.0146148 (PMC4703409; doi:10.1371/journal.pone.0146148)
Supplement: S2 Fig — (PDF) [file pone.0146148.s002.pdf]

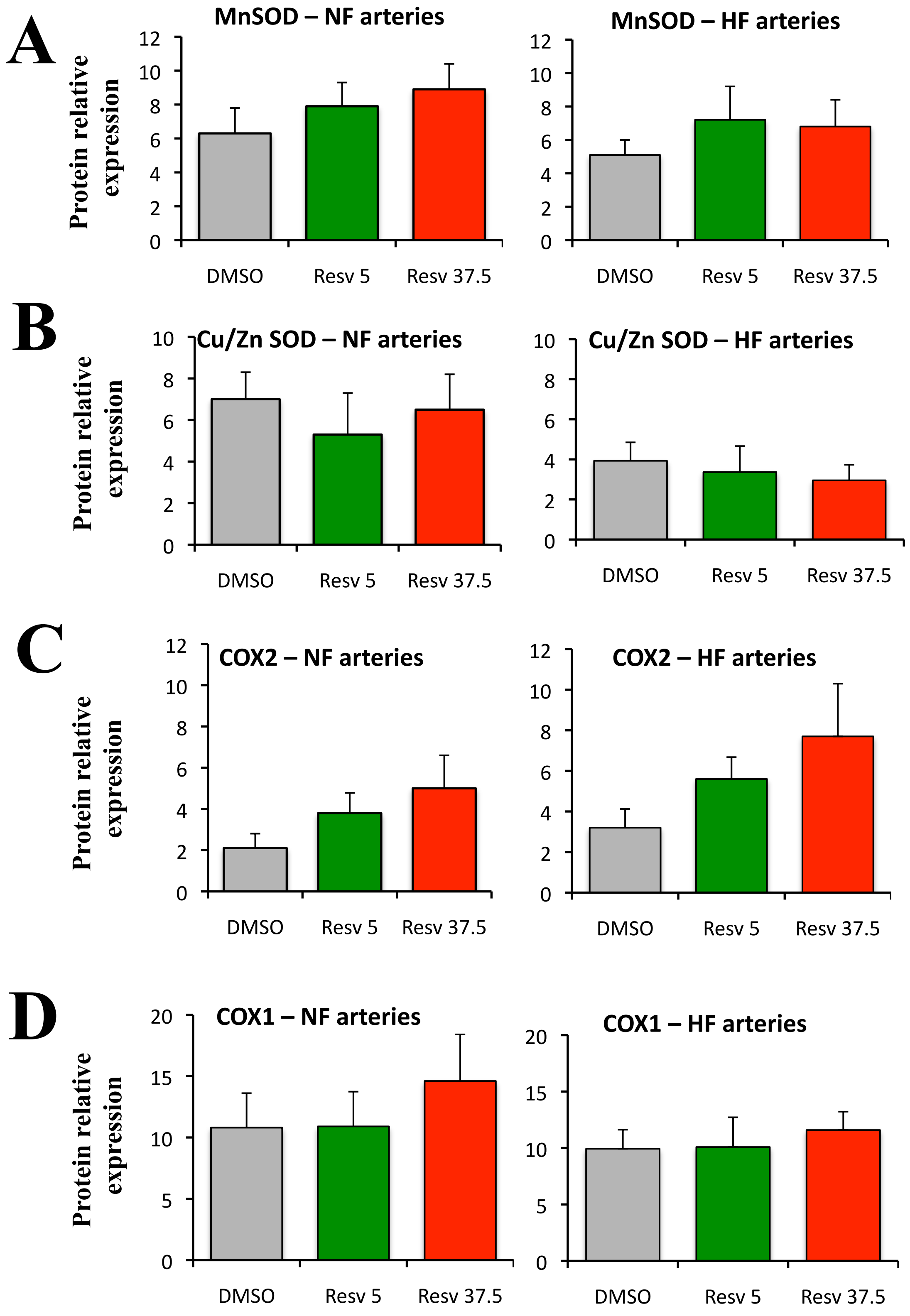

S2 Fig. : SOD and COX expression level: The expression level of MnSOD (A), Cu/ZnSOD (B), COX2 (C) and COX1 (D) was determined using Western-blot analysis in HF (right panel) and NF arteries (left panel). Arteries were isolated from ovariectomized rats treated with resveratrol 5.0 (R5, n=9 rats) or 37.5mg/kg (R37.5, n=9 rats) or with the vehicle (DMSO, n=10 rats). Mean ± sem is represented. NS, effect of the treatments (versus DMSO).
