# Supplementary material for: Resveratrol Improved Flow-Mediated Outward Arterial Remodeling in Ovariectomized Rats with Hypertrophic Effect at High Dose
Source: PLoS One. 2016 Jan 6;11(1):e0146148. doi: 10.1371/journal.pone.0146148 (PMC4703409; doi:10.1371/journal.pone.0146148)
Supplement: S3 Fig — (PDF) [file pone.0146148.s003.pdf]

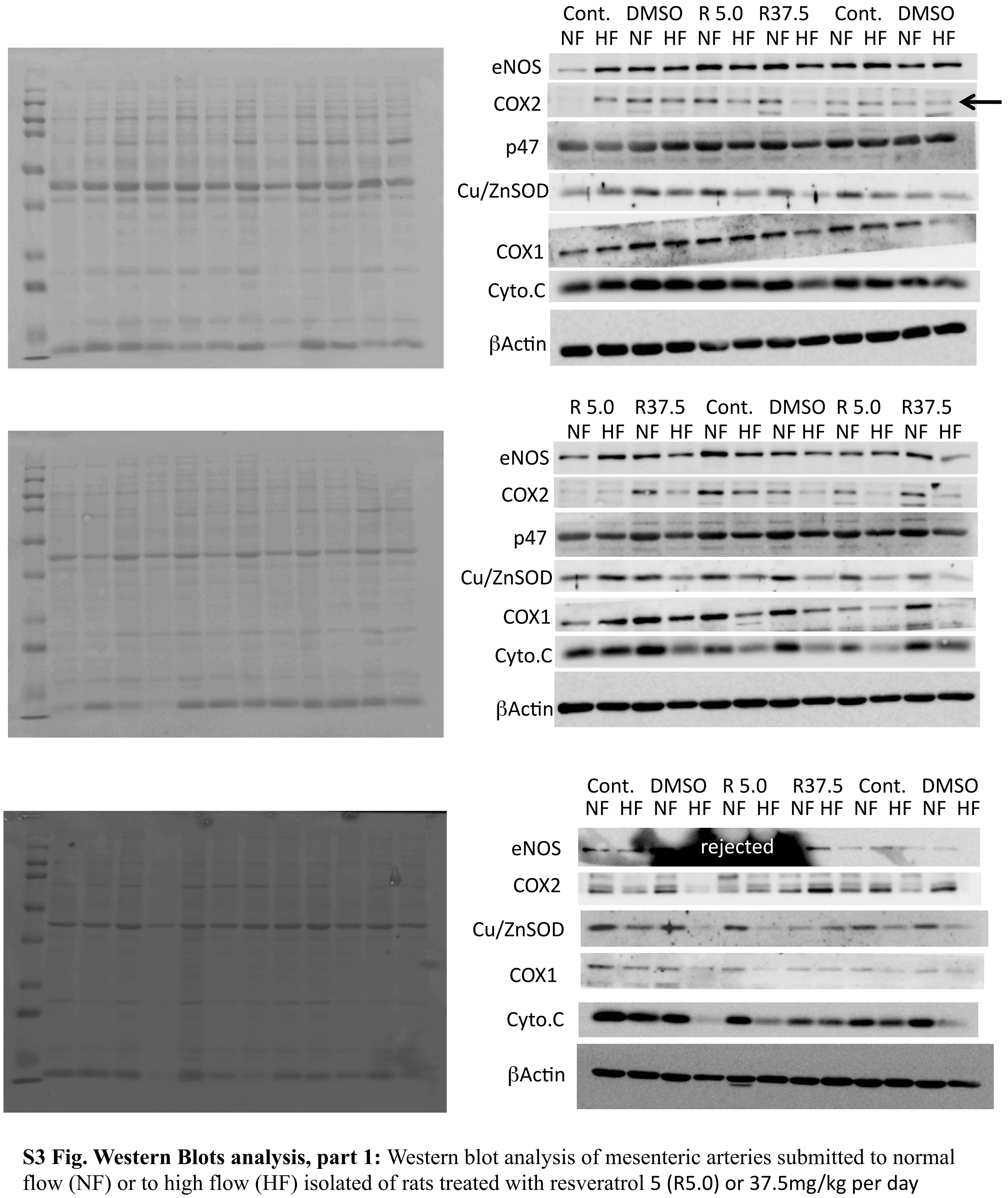

S3 Fig. Western Blots analysis, part 1: Western blot analysis of mesenteric arteries submitted to normal flow (NF) or to high flow (HF) isolated of rats treated with resveratrol 5 (R5.0) or 37.5mg/kg per day (R37.5) or with the solvent (DMSO) or nothing (Cont.).
The following proteins were analyzed: endothelial NO syntase (eNOS), cyclooxygenase-2 (COX2) , p47phox, Cu/ZnSOD, cyclooxygenase-1 (COX1), Cytochrome C Oxidase IV (Cyto C) and beta actin (βactin)

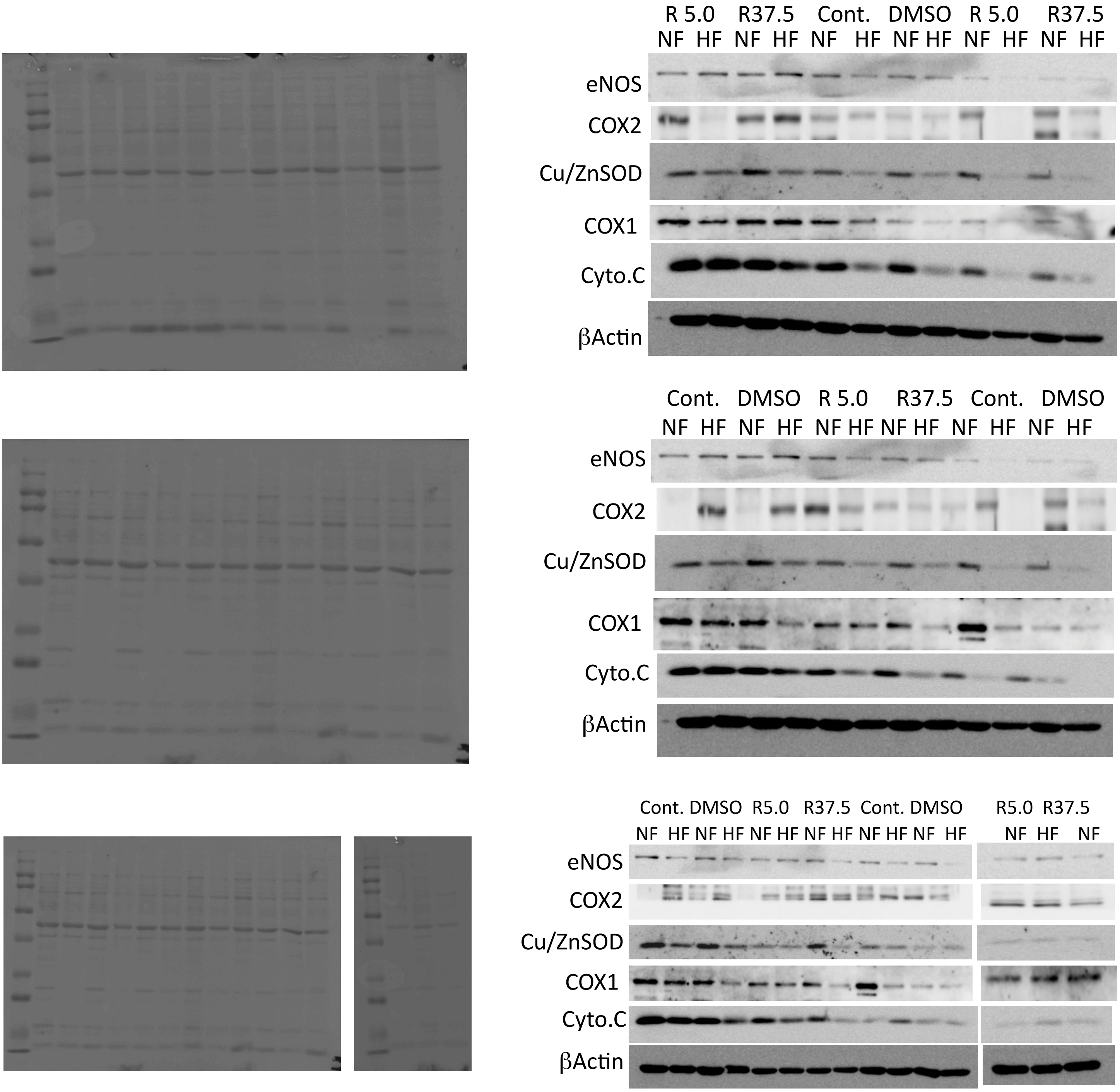

S3 Fig. Western Blots analysis, part 2: Western blot analysis of mesenteric arteries submitted to normal flow (NF) or to high flow (HF) isolated of rats treated with resveratrol 5 (R5.0) or 37.5mg/kg per day (R37.5) or with the solvent (DMSO) or nothing (Cont.).

The following proteins were analyzed: endothelial NO syntase (eNOS), cyclooxygenase-2 (COX2) , p47phox, Cu/ZnSOD, cyclooxygenase-1 (COX1), Cytochrome C Oxidase IV (Cyto C) and beta actin (βactin)

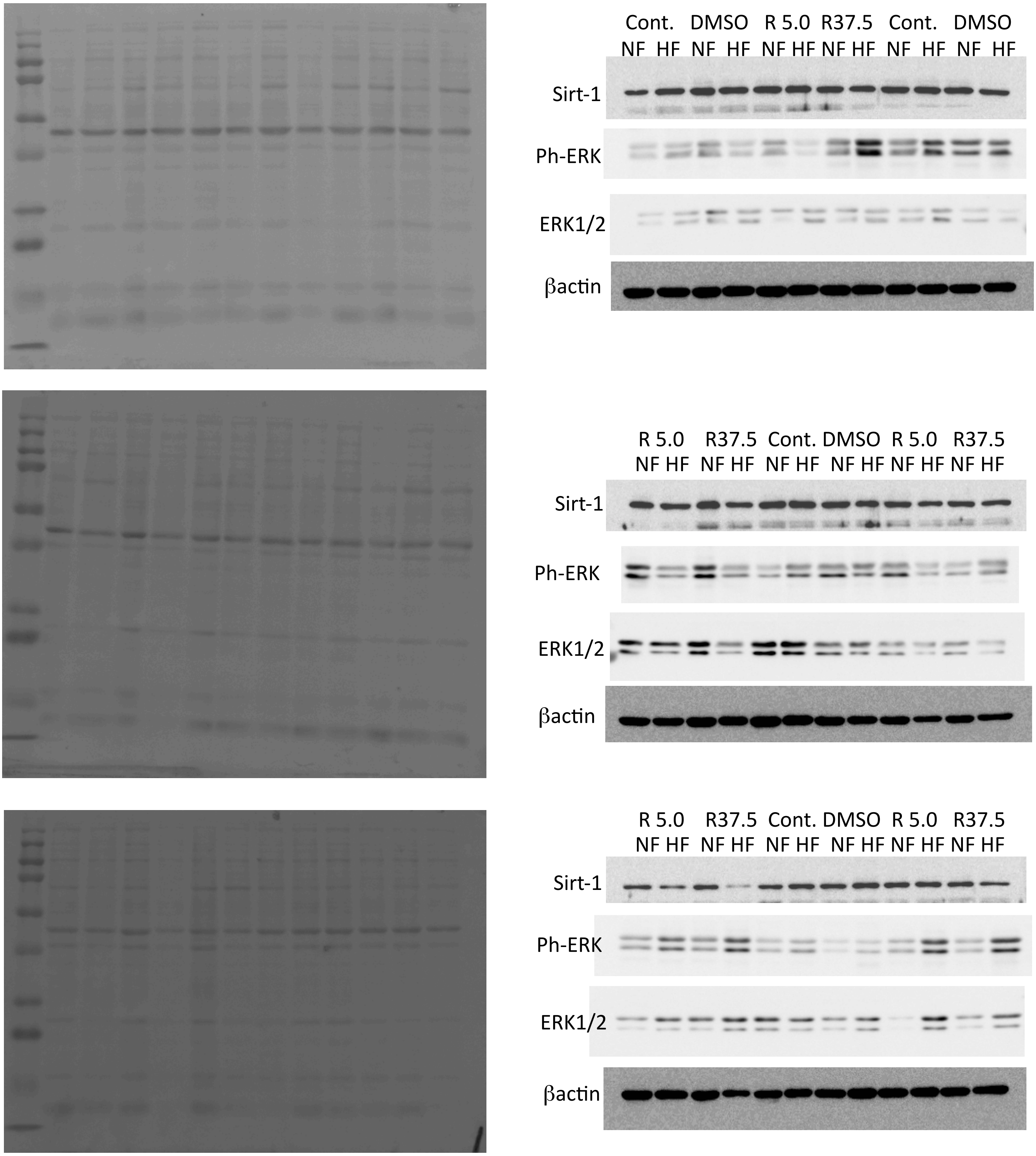

S3 Fig. Western Blots analysis, part 3: Western blot analysis of mesenteric arteries submitted to normal flow (NF) or to high flow (HF) isolated of rats treated with resveratrol 5 (R5.0) or 37.5mg/kg per day (R37.5) or with the solvent (DMSO) or nothing (Cont.).

The following proteins were analyzed: Sirtuin-1 (Sirt-1), phospho-ERK1/2 (ph-ERK), ERK1/2 and beta actin (βactin)

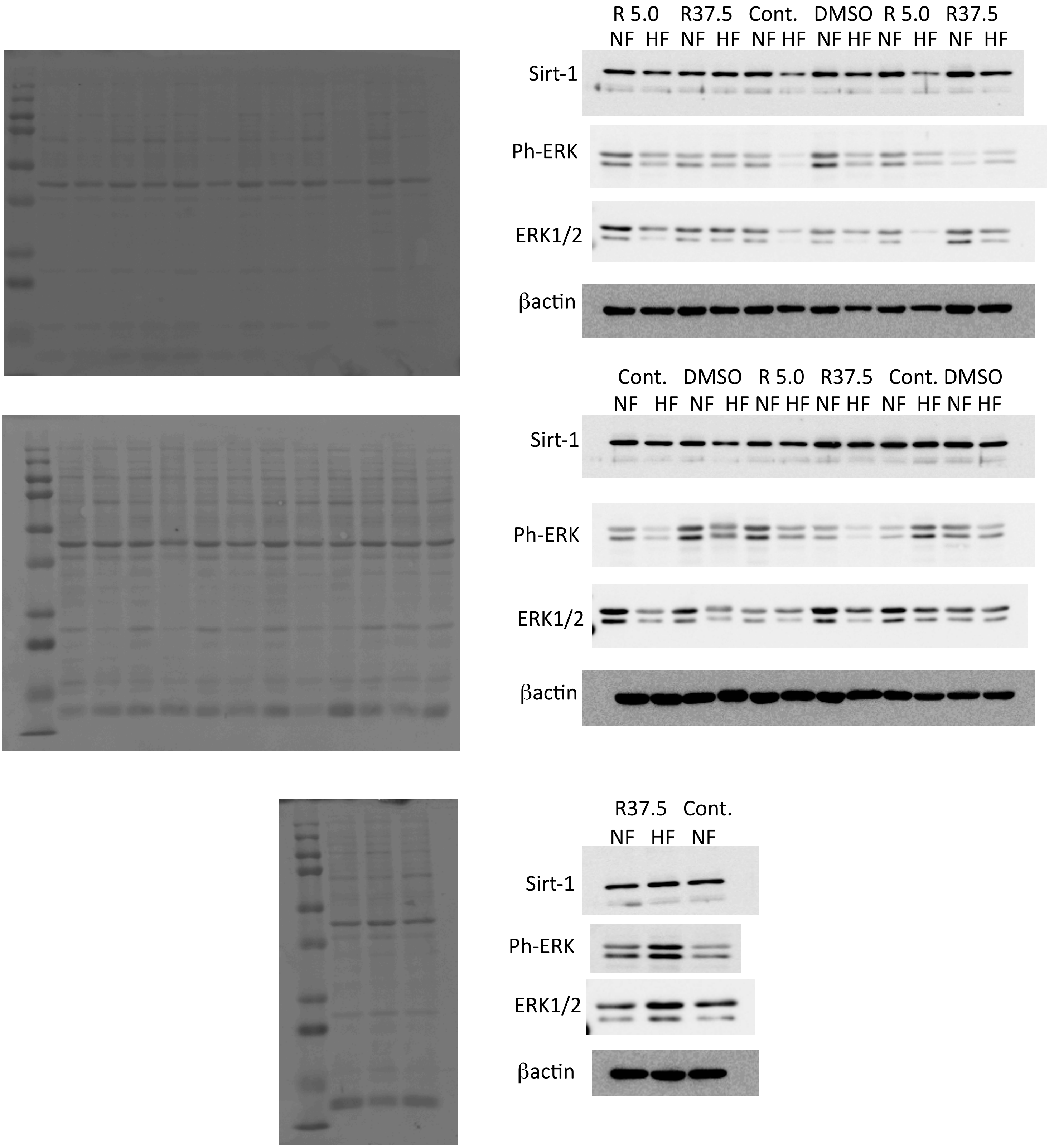

S3 Fig. Western Blots analysis, part 4: Western blot analysis of mesenteric arteries submitted to normal flow (NF) or to high flow (HF) isolated of rats treated with resveratrol 5 (R5.0) or 37.5mg/kg per day (R37.5) or with the solvent (DMSO) or nothing (Cont.).
The following proteins were analyzed: Sirtuin-1 (Sirt-1), phospho-ERK1/2 (ph-ERK), ERK1/2 and beta actin (βactin)
